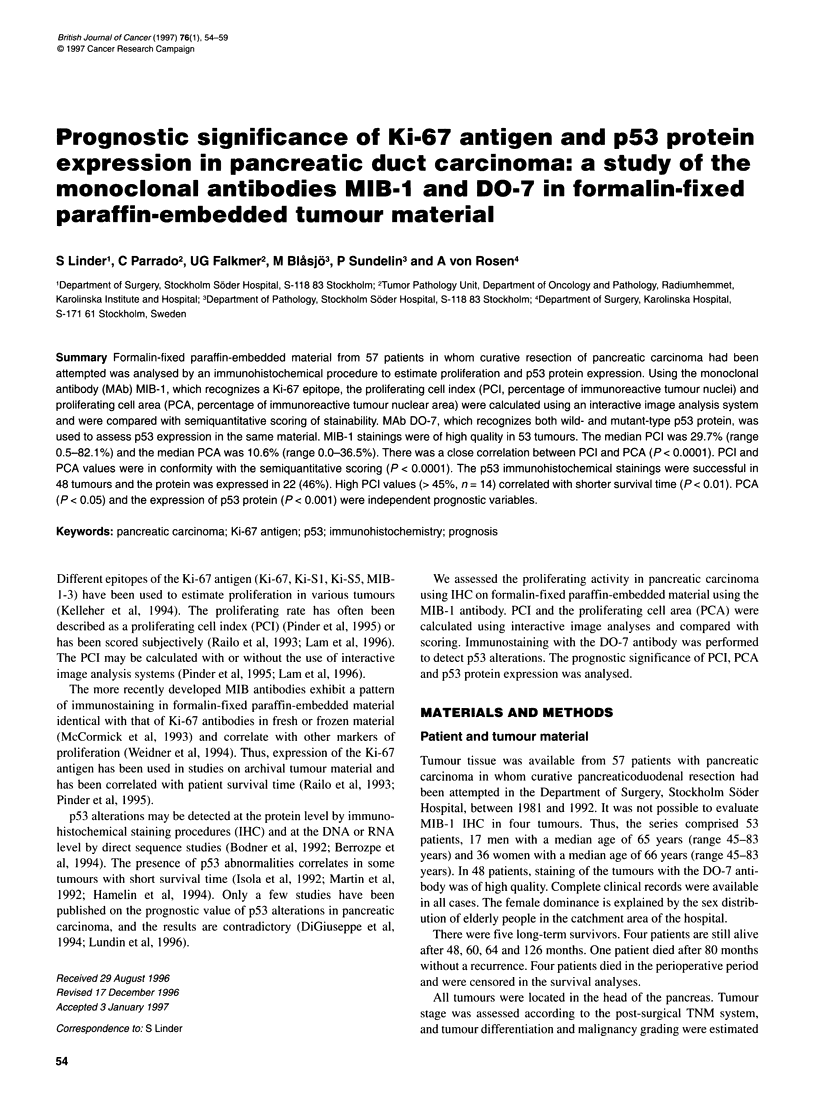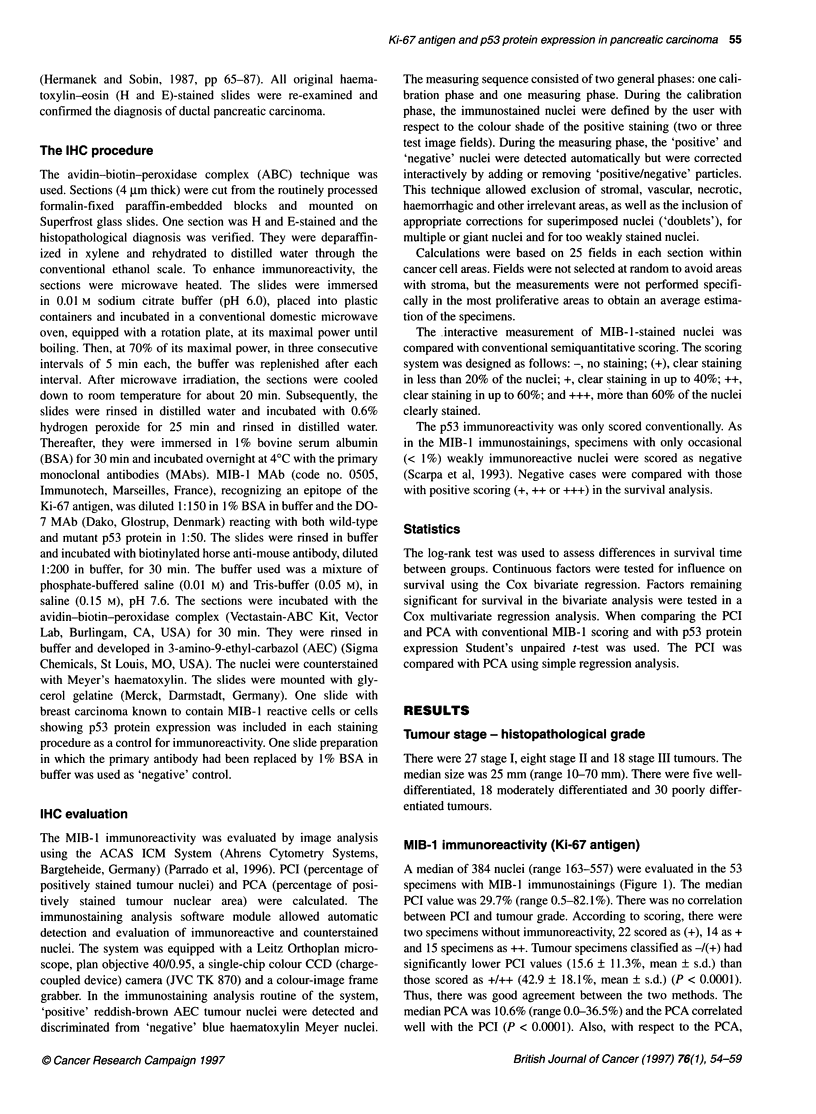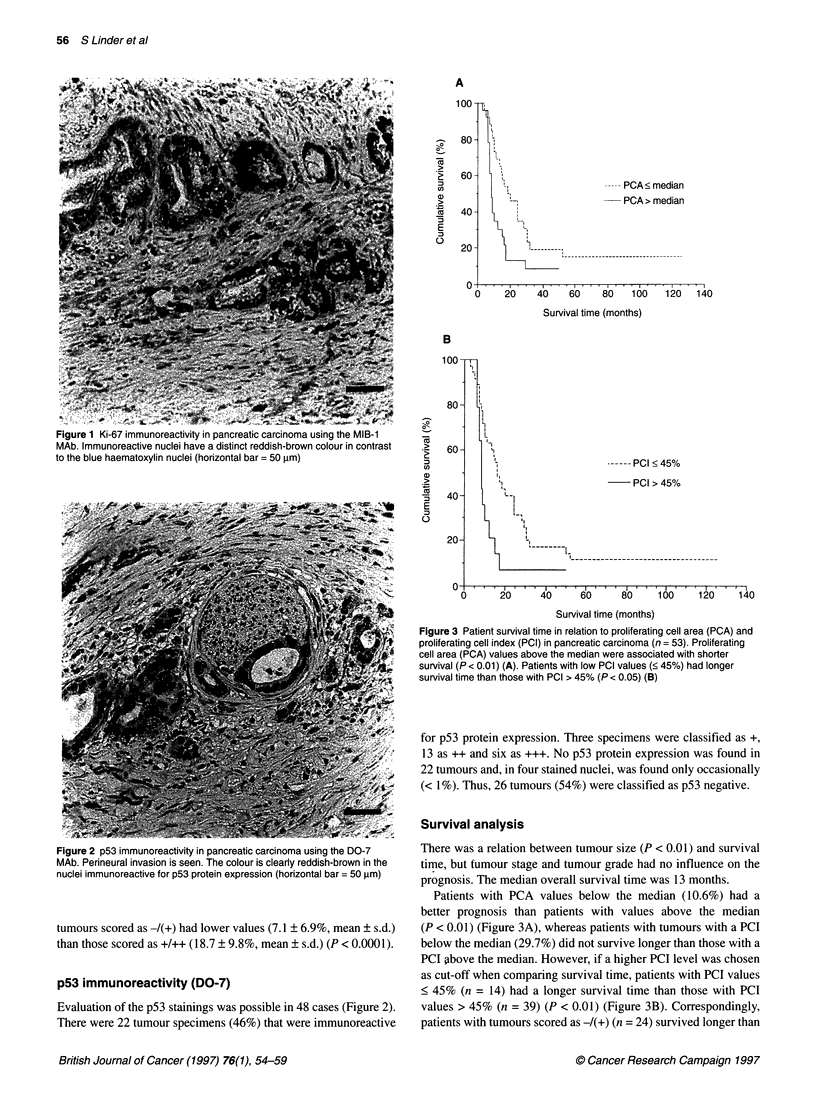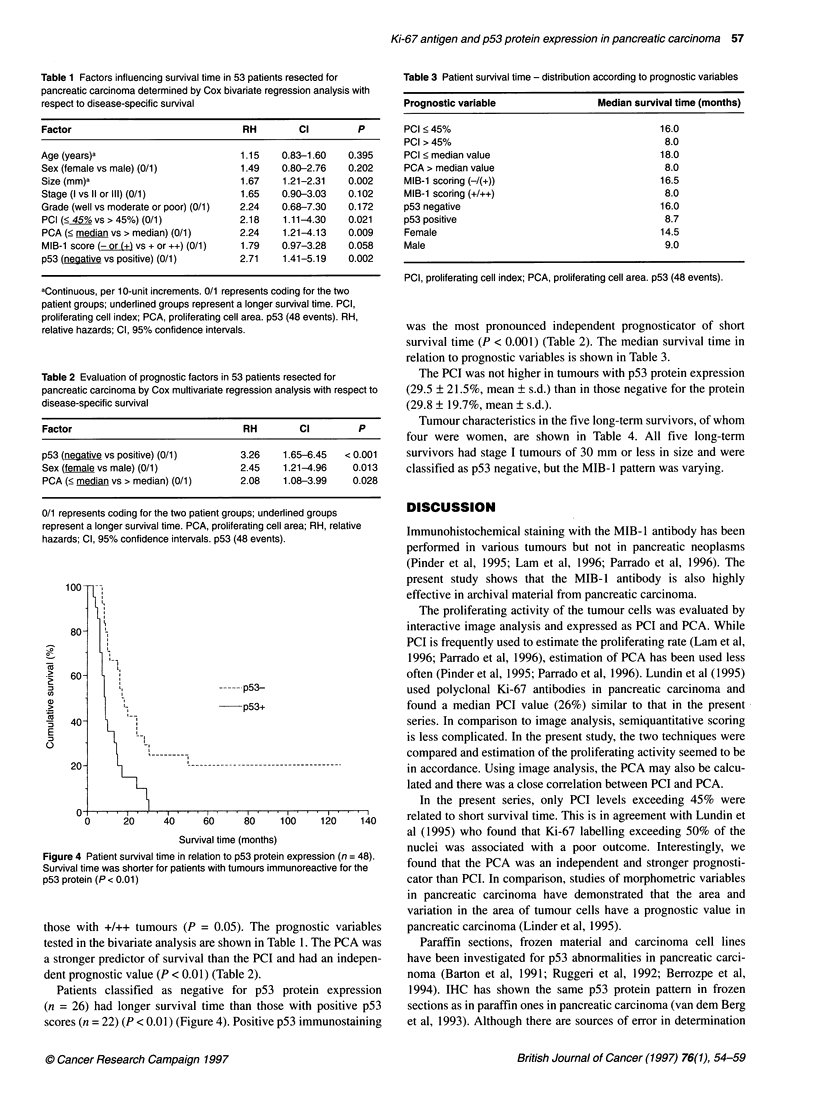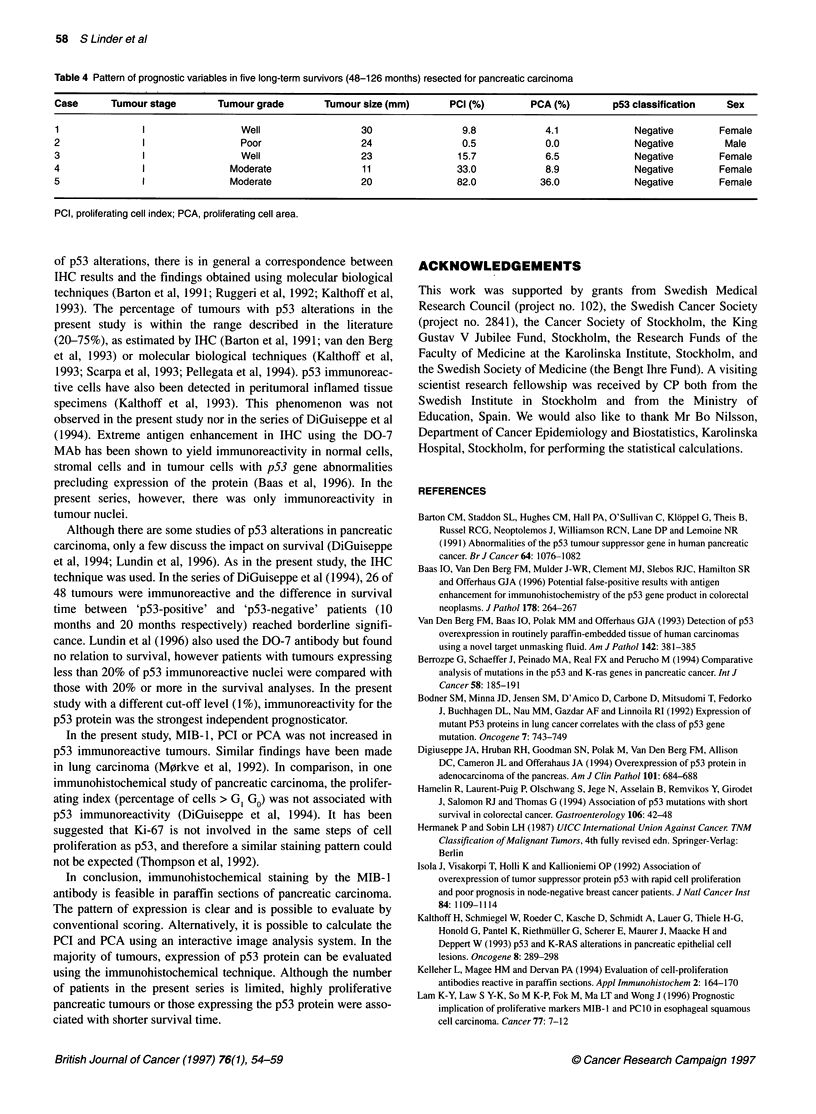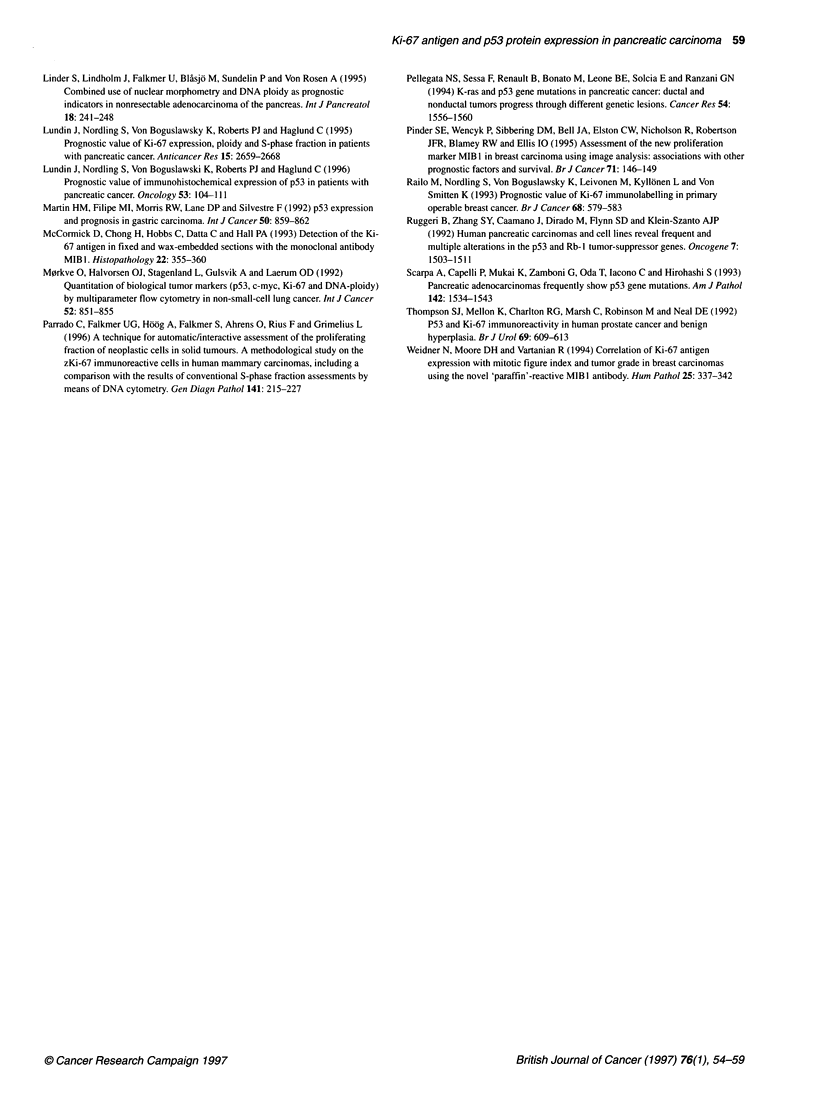# Prognostic significance of Ki-67 antigen and p53 protein expression in pancreatic duct carcinoma: a study of the monoclonal antibodies MIB-1 and DO-7 in formalin-fixed paraffin-embedded tumour material.

**DOI:** 10.1038/bjc.1997.336

**Published:** 1997

**Authors:** S. Linder, C. Parrado, U. G. Falkmer, M. BlÃ¥sjÃ¶, P. Sundelin, A. von Rosen

**Affiliations:** Department of Surgery, Stockholm SÃ¶der Hospital, Sweden.

## Abstract

**Images:**


*British Journal of Cancer* (1997) **76**(1), 54–59
© 1997 Cancer Research Campaign

### Prognostic significance of Ki-67 antigen and p53 protein expression in pancreatic duct carcinoma: a study of the monoclonal antibodies MIB-1 and DO-7 in formalin-fixed paraffin-embedded tumour material

**S Linder[1], C Parrado[2], UG Falkmer[2], M Blåsjö[3], P Sundelin[3] and A von Rosen[4]**

[1]Department of Surgery, Stockholm Söder Hospital, S-118 83 Stockholm; [2]Tumor Pathology Unit, Department of Oncology and Pathology, Radiumhemmet, Karolinska Institute and Hospital; [3]Department of Pathology, Stockholm Söder Hospital, S-118 83 Stockholm; [4]Department of Surgery, Karolinska Hospital, S-171 61 Stockholm, Sweden

**Summary** Formalin-fixed paraffin-embedded material from 57 patients in whom curative resection of pancreatic carcinoma had been attempted was analysed by an immunohistochemical procedure to estimate proliferation and p53 protein expression. Using the monoclonal antibody (MAb) MIB-1, which recognizes a Ki-67 epitope, the proliferating cell index (PCI, percentage of immunoreactive tumour nuclei) and proliferating cell area (PCA, percentage of immunoreactive tumour nuclear area) were calculated using an interactive image analysis system and were compared with semiquantitative scoring of stainability. MAb DO-7, which recognizes both wild- and mutant-type p53 protein, was used to assess p53 expression in the same material. MIB-1 stainings were of high quality in 53 tumours. The median PCI was 29.7% (range 0.5–82.1%) and the median PCA was 10.6% (range 0.0–36.5%). There was a close correlation between PCI and PCA ($P < 0.0001$). PCI and PCA values were in conformity with the semiquantitative scoring ($P < 0.0001$). The p53 immunohistochemical stainings were successful in 48 tumours and the protein was expressed in 22 (46%). High PCI values (> 45%, $n = 14$) correlated with shorter survival time ($P < 0.01$). PCA ($P < 0.05$) and the expression of p53 protein ($P < 0.001$) were independent prognostic variables.

**Keywords:** pancreatic carcinoma; Ki-67 antigen; p53; immunohistochemistry; prognosis

Different epitopes of the Ki-67 antigen (Ki-67, Ki-S1, Ki-S5, MIB-1-3) have been used to estimate proliferation in various tumours (Kelleher et al, 1994). The proliferating rate has often been described as a proliferating cell index (PCI) (Pinder et al, 1995) or has been scored subjectively (Railo et al, 1993; Lam et al, 1996). The PCI may be calculated with or without the use of interactive image analysis systems (Pinder et al, 1995; Lam et al, 1996).

The more recently developed MIB antibodies exhibit a pattern of immunostaining in formalin-fixed paraffin-embedded material identical with that of Ki-67 antibodies in fresh or frozen material (McCormick et al, 1993) and correlate with other markers of proliferation (Weidner et al, 1994). Thus, expression of the Ki-67 antigen has been used in studies on archival tumour material and has been correlated with patient survival time (Railo et al, 1993; Pinder et al, 1995).

p53 alterations may be detected at the protein level by immunohistochemical staining procedures (IHC) and at the DNA or RNA level by direct sequence studies (Bodner et al, 1992; Berrozpe et al, 1994). The presence of p53 abnormalities correlates in some tumours with short survival time (Isola et al, 1992; Martin et al, 1992; Hamelin et al, 1994). Only a few studies have been published on the prognostic value of p53 alterations in pancreatic carcinoma, and the results are contradictory (DiGiuseppe et al, 1994; Lundin et al, 1996).

*Received 29 August 1996*
*Revised 17 December 1996*
*Accepted 3 January 1997*

*Correspondence to:* S Linder

We assessed the proliferating activity in pancreatic carcinoma using IHC on formalin-fixed paraffin-embedded material using the MIB-1 antibody. PCI and the proliferating cell area (PCA) were calculated using interactive image analyses and compared with scoring. Immunostaining with the DO-7 antibody was performed to detect p53 alterations. The prognostic significance of PCI, PCA and p53 protein expression was analysed.

### MATERIALS AND METHODS

### Patient and tumour material

Tumour tissue was available from 57 patients with pancreatic carcinoma in whom curative pancreaticoduodenal resection had been attempted in the Department of Surgery, Stockholm Söder Hospital, between 1981 and 1992. It was not possible to evaluate MIB-1 IHC in four tumours. Thus, the series comprised 53 patients, 17 men with a median age of 65 years (range 45–83 years) and 36 women with a median age of 66 years (range 45–83 years). In 48 patients, staining of the tumours with the DO-7 antibody was of high quality. Complete clinical records were available in all cases. The female dominance is explained by the sex distribution of elderly people in the catchment area of the hospital.

There were five long-term survivors. Four patients are still alive after 48, 60, 64 and 126 months. One patient died after 80 months without a recurrence. Four patients died in the perioperative period and were censored in the survival analyses.

All tumours were located in the head of the pancreas. Tumour stage was assessed according to the post-surgical TNM system, and tumour differentiation and malignancy grading were estimated

(Hermanek and Sobin, 1987, pp 65–87). All original haematoxylin–eosin (H and E)-stained slides were re-examined and confirmed the diagnosis of ductal pancreatic carcinoma.

### The IHC procedure

The avidin–biotin–peroxidase complex (ABC) technique was used. Sections (4 μm thick) were cut from the routinely processed formalin-fixed paraffin-embedded blocks and mounted on Superfrost glass slides. One section was H and E-stained and the histopathological diagnosis was verified. They were deparaffinized in xylene and rehydrated to distilled water through the conventional ethanol scale. To enhance immunoreactivity, the sections were microwave heated. The slides were immersed in 0.01 M sodium citrate buffer (pH 6.0), placed into plastic containers and incubated in a conventional domestic microwave oven, equipped with a rotation plate, at its maximal power until boiling. Then, at 70% of its maximal power, in three consecutive intervals of 5 min each, the buffer was replenished after each interval. After microwave irradiation, the sections were cooled down to room temperature for about 20 min. Subsequently, the slides were rinsed in distilled water and incubated with 0.6% hydrogen peroxide for 25 min and rinsed in distilled water. Thereafter, they were immersed in 1% bovine serum albumin (BSA) for 30 min and incubated overnight at 4°C with the primary monoclonal antibodies (MAbs). MIB-1 MAb (code no. 0505, Immunotech, Marseilles, France), recognizing an epitope of the Ki-67 antigen, was diluted 1:150 in 1% BSA in buffer and the DO-7 MAb (Dako, Glostrup, Denmark) reacting with both wild-type and mutant p53 protein in 1:50. The slides were rinsed in buffer and incubated with biotinylated horse anti-mouse antibody, diluted 1:200 in buffer, for 30 min. The buffer used was a mixture of phosphate-buffered saline (0.01 M) and Tris-buffer (0.05 M), in saline (0.15 M), pH 7.6. The sections were incubated with the avidin–biotin–peroxidase complex (Vectastain-ABC Kit, Vector Lab, Burlingam, CA, USA) for 30 min. They were rinsed in buffer and developed in 3-amino-9-ethyl-carbazol (AEC) (Sigma Chemicals, St Louis, MO, USA). The nuclei were counterstained with Meyer's haematoxylin. The slides were mounted with glycerol gelatine (Merck, Darmstadt, Germany). One slide with breast carcinoma known to contain MIB-1 reactive cells or cells showing p53 protein expression was included in each staining procedure as a control for immunoreactivity. One slide preparation in which the primary antibody had been replaced by 1% BSA in buffer was used as 'negative' control.

### IHC evaluation

The MIB-1 immunoreactivity was evaluated by image analysis using the ACAS ICM System (Ahrens Cytometry Systems, Bargteheide, Germany) (Parrado et al, 1996). PCI (percentage of positively stained tumour nuclei) and PCA (percentage of positively stained tumour nuclear area) were calculated. The immunostaining analysis software module allowed automatic detection and evaluation of immunoreactive and counterstained nuclei. The system was equipped with a Leitz Orthoplan microscope, plan objective 40/0.95, a single-chip colour CCD (charge-coupled device) camera (JVC TK 870) and a colour-image frame grabber. In the immunostaining analysis routine of the system, 'positive' reddish-brown AEC tumour nuclei were detected and discriminated from 'negative' blue haematoxylin Meyer nuclei. The measuring sequence consisted of two general phases: one calibration phase and one measuring phase. During the calibration phase, the immunostained nuclei were defined by the user with respect to the colour shade of the positive staining (two or three test image fields). During the measuring phase, the 'positive' and 'negative' nuclei were detected automatically but were corrected interactively by adding or removing 'positive/negative' particles. This technique allowed exclusion of stromal, vascular, necrotic, haemorrhagic and other irrelevant areas, as well as the inclusion of appropriate corrections for superimposed nuclei ('doublets'), for multiple or giant nuclei and for too weakly stained nuclei.

Calculations were based on 25 fields in each section within cancer cell areas. Fields were not selected at random to avoid areas with stroma, but the measurements were not performed specifically in the most proliferative areas to obtain an average estimation of the specimens.

The interactive measurement of MIB-1-stained nuclei was compared with conventional semiquantitative scoring. The scoring system was designed as follows: –, no staining; (+), clear staining in less than 20% of the nuclei; +, clear staining in up to 40%; ++, clear staining in up to 60%; and +++, more than 60% of the nuclei clearly stained.

The p53 immunoreactivity was only scored conventionally. As in the MIB-1 immunostainings, specimens with only occasional (< 1%) weakly immunoreactive nuclei were scored as negative (Scarpa et al, 1993). Negative cases were compared with those with positive scoring (+, ++ or +++) in the survival analysis.

### Statistics

The log-rank test was used to assess differences in survival time between groups. Continuous factors were tested for influence on survival using the Cox bivariate regression. Factors remaining significant for survival in the bivariate analysis were tested in a Cox multivariate regression analysis. When comparing the PCI and PCA with conventional MIB-1 scoring and with p53 protein expression Student's unpaired *t*-test was used. The PCI was compared with PCA using simple regression analysis.

### RESULTS

### Tumour stage – histopathological grade

There were 27 stage I, eight stage II and 18 stage III tumours. The median size was 25 mm (range 10–70 mm). There were five well-differentiated, 18 moderately differentiated and 30 poorly differentiated tumours.

### MIB-1 immunoreactivity (Ki-67 antigen)

A median of 384 nuclei (range 163–557) were evaluated in the 53 specimens with MIB-1 immunostainings (Figure 1). The median PCI value was 29.7% (range 0.5–82.1%). There was no correlation between PCI and tumour grade. According to scoring, there were two specimens without immunoreactivity, 22 scored as (+), 14 as + and 15 specimens as ++. Tumour specimens classified as –/(+) had significantly lower PCI values (15.6 ± 11.3%, mean ± s.d.) than those scored as +/++ (42.9 ± 18.1%, mean ± s.d.) ($P < 0.0001$). Thus, there was good agreement between the two methods. The median PCA was 10.6% (range 0.0–36.5%) and the PCA correlated well with the PCI ($P < 0.0001$). Also, with respect to the PCA,

© Cancer Research Campaign 1997

Figure 1 Ki-67 immunoreactivity in pancreatic carcinoma using the MIB-1 MAb. Immunoreactive nuclei have a distinct reddish-brown colour in contrast to the blue haematoxylin nuclei (horizontal bar = 50 μm)

Figure 2 p53 immunoreactivity in pancreatic carcinoma using the DO-7 MAb. Perineural invasion is seen. The colour is clearly reddish-brown in the nuclei immunoreactive for p53 protein expression (horizontal bar = 50 μm)

tumours scored as –/(+) had lower values (7.1 ± 6.9%, mean ± s.d.) than those scored as +/++ (18.7 ± 9.8%, mean ± s.d.) ($P < 0.0001$).

### p53 immunoreactivity (DO-7)

Evaluation of the p53 stainings was possible in 48 cases (Figure 2). There were 22 tumour specimens (46%) that were immunoreactive for p53 protein expression. Three specimens were classified as +, 13 as ++ and six as +++. No p53 protein expression was found in 22 tumours and, in four stained nuclei, was found only occasionally (< 1%). Thus, 26 tumours (54%) were classified as p53 negative.

Figure 3 Patient survival time in relation to proliferating cell area (PCA) and proliferating cell index (PCI) in pancreatic carcinoma ($n$ = 53). Proliferating cell area (PCA) values above the median were associated with shorter survival ($P < 0.01$) (**A**). Patients with low PCI values (≤ 45%) had longer survival time than those with PCI > 45% ($P < 0.05$) (**B**)

### Survival analysis

There was a relation between tumour size ($P < 0.01$) and survival time, but fumour stage and tumour grade had no influence on the prognosis. The median overall survival time was 13 months.

Patients with PCA values below the median (10.6%) had a better prognosis than patients with values above the median ($P < 0.01$) (Figure 3A), whereas patients with tumours with a PCI below the median (29.7%) did not survive longer than those with a PCI above the median. However, if a higher PCI level was chosen as cut-off when comparing survival time, patients with PCI values ≤ 45% ($n$ = 14) had a longer survival time than those with PCI values > 45% ($n$ = 39) ($P < 0.01$) (Figure 3B). Correspondingly, patients with tumours scored as –/(+) ($n$ = 24) survived longer than

© Cancer Research Campaign 1997

**Table 1** Factors influencing survival time in 53 patients resected for pancreatic carcinoma determined by Cox bivariate regression analysis with respect to disease-specific survival

| Factor | RH | CI | *P* |
|---|---|---|---|
| Age (years)[a] | 1.15 | 0.83–1.60 | 0.395 |
| Sex (female vs male) (0/1) | 1.49 | 0.80–2.76 | 0.202 |
| Size (mm)[a] | 1.67 | 1.21–2.31 | 0.002 |
| Stage (I vs II or III) (0/1) | 1.65 | 0.90–3.03 | 0.102 |
| Grade (well vs moderate or poor) (0/1) | 2.24 | 0.68–7.30 | 0.172 |
| PCI (≤ 45% vs > 45%) (0/1) | 2.18 | 1.11–4.30 | 0.021 |
| PCA (≤ median vs > median) (0/1) | 2.24 | 1.21–4.13 | 0.009 |
| MIB-1 score (– or (+) vs + or ++) (0/1) | 1.79 | 0.97–3.28 | 0.058 |
| p53 (negative vs positive) (0/1) | 2.71 | 1.41–5.19 | 0.002 |

[a]Continuous, per 10-unit increments. 0/1 represents coding for the two patient groups; underlined groups represent a longer survival time. PCI, proliferating cell index; PCA, proliferating cell area. p53 (48 events). RH, relative hazards; CI, 95% confidence intervals.

**Table 2** Evaluation of prognostic factors in 53 patients resected for pancreatic carcinoma by Cox multivariate regression analysis with respect to disease-specific survival

| Factor | RH | CI | *P* |
|---|---|---|---|
| p53 (negative vs positive) (0/1) | 3.26 | 1.65–6.45 | < 0.001 |
| Sex (female vs male) (0/1) | 2.45 | 1.21–4.96 | 0.013 |
| PCA (≤ median vs > median) (0/1) | 2.08 | 1.08–3.99 | 0.028 |

0/1 represents coding for the two patient groups; underlined groups represent a longer survival time. PCA, proliferating cell area; RH, relative hazards; CI, 95% confidence intervals. p53 (48 events).

**Figure 4** Patient survival time in relation to p53 protein expression ($n$ = 48). Survival time was shorter for patients with tumours immunoreactive for the p53 protein ($P < 0.01$)

those with +/++ tumours ($P$ = 0.05). The prognostic variables tested in the bivariate analysis are shown in Table 1. The PCA was a stronger predictor of survival than the PCI and had an independent prognostic value ($P < 0.01$) (Table 2).

Patients classified as negative for p53 protein expression ($n$ = 26) had longer survival time than those with positive p53 scores ($n$ = 22) ($P < 0.01$) (Figure 4). Positive p53 immunostaining

**Table 3** Patient survival time – distribution according to prognostic variables

| Prognostic variable | Median survival time (months) |
|---|---|
| PCI ≤ 45% | 16.0 |
| PCI > 45% | 8.0 |
| PCI ≤ median value | 18.0 |
| PCA > median value | 8.0 |
| MIB-1 scoring (–/(+)) | 16.5 |
| MIB-1 scoring (+/++) | 8.0 |
| p53 negative | 16.0 |
| p53 positive | 8.7 |
| Female | 14.5 |
| Male | 9.0 |

PCI, proliferating cell index; PCA, proliferating cell area. p53 (48 events).

was the most pronounced independent prognosticator of short survival time ($P < 0.001$) (Table 2). The median survival time in relation to prognostic variables is shown in Table 3.

The PCI was not higher in tumours with p53 protein expression (29.5 ± 21.5%, mean ± s.d.) than in those negative for the protein (29.8 ± 19.7%, mean ± s.d.).

Tumour characteristics in the five long-term survivors, of whom four were women, are shown in Table 4. All five long-term survivors had stage I tumours of 30 mm or less in size and were classified as p53 negative, but the MIB-1 pattern was varying.

### DISCUSSION

Immunohistochemical staining with the MIB-1 antibody has been performed in various tumours but not in pancreatic neoplasms (Pinder et al, 1995; Lam et al, 1996; Parrado et al, 1996). The present study shows that the MIB-1 antibody is also highly effective in archival material from pancreatic carcinoma.

The proliferating activity of the tumour cells was evaluated by interactive image analysis and expressed as PCI and PCA. While PCI is frequently used to estimate the proliferating rate (Lam et al, 1996; Parrado et al, 1996), estimation of PCA has been used less often (Pinder et al, 1995; Parrado et al, 1996). Lundin et al (1995) used polyclonal Ki-67 antibodies in pancreatic carcinoma and found a median PCI value (26%) similar to that in the present series. In comparison to image analysis, semiquantitative scoring is less complicated. In the present study, the two techniques were compared and estimation of the proliferating activity seemed to be in accordance. Using image analysis, the PCA may also be calculated and there was a close correlation between PCI and PCA.

In the present series, only PCI levels exceeding 45% were related to short survival time. This is in agreement with Lundin et al (1995) who found that Ki-67 labelling exceeding 50% of the nuclei was associated with a poor outcome. Interestingly, we found that the PCA was an independent and stronger prognosticator than PCI. In comparison, studies of morphometric variables in pancreatic carcinoma have demonstrated that the area and variation in the area of tumour cells have a prognostic value in pancreatic carcinoma (Linder et al, 1995).

Paraffin sections, frozen material and carcinoma cell lines have been investigated for p53 abnormalities in pancreatic carcinoma (Barton et al, 1991; Ruggeri et al, 1992; Berrozpe et al, 1994). IHC has shown the same p53 protein pattern in frozen sections as in paraffin ones in pancreatic carcinoma (van dem Berg et al, 1993). Although there are sources of error in determination

© Cancer Research Campaign 1997

Table 4 Pattern of prognostic variables in five long-term survivors (48–126 months) resected for pancreatic carcinoma

| Case | Tumour stage | Tumour grade | Tumour size (mm) | PCI (%) | PCA (%) | p53 classification | Sex |
|---|---|---|---|---|---|---|---|
| 1 | I | Well | 30 | 9.8 | 4.1 | Negative | Female |
| 2 | I | Poor | 24 | 0.5 | 0.0 | Negative | Male |
| 3 | I | Well | 23 | 15.7 | 6.5 | Negative | Female |
| 4 | I | Moderate | 11 | 33.0 | 8.9 | Negative | Female |
| 5 | I | Moderate | 20 | 82.0 | 36.0 | Negative | Female |

PCI, proliferating cell index; PCA, proliferating cell area.

of p53 alterations, there is in general a correspondence between IHC results and the findings obtained using molecular biological techniques (Barton et al, 1991; Ruggeri et al, 1992; Kalthoff et al, 1993). The percentage of tumours with p53 alterations in the present study is within the range described in the literature (20–75%), as estimated by IHC (Barton et al, 1991; van den Berg et al, 1993) or molecular biological techniques (Kalthoff et al, 1993; Scarpa et al, 1993; Pellegata et al, 1994). p53 immunoreactive cells have also been detected in peritumoral inflamed tissue specimens (Kalthoff et al, 1993). This phenomenon was not observed in the present study nor in the series of DiGuiseppe et al (1994). Extreme antigen enhancement in IHC using the DO-7 MAb has been shown to yield immunoreactivity in normal cells, stromal cells and in tumour cells with *p53* gene abnormalities precluding expression of the protein (Baas et al, 1996). In the present series, however, there was only immunoreactivity in tumour nuclei.

Although there are some studies of p53 alterations in pancreatic carcinoma, only a few discuss the impact on survival (DiGuiseppe et al, 1994; Lundin et al, 1996). As in the present study, the IHC technique was used. In the series of DiGuiseppe et al (1994), 26 of 48 tumours were immunoreactive and the difference in survival time between 'p53-positive' and 'p53-negative' patients (10 months and 20 months respectively) reached borderline significance. Lundin et al (1996) also used the DO-7 antibody but found no relation to survival, however patients with tumours expressing less than 20% of p53 immunoreactive nuclei were compared with those with 20% or more in the survival analyses. In the present study with a different cut-off level (1%), immunoreactivity for the p53 protein was the strongest independent prognosticator.

In the present study, MIB-1, PCI or PCA was not increased in p53 immunoreactive tumours. Similar findings have been made in lung carcinoma (Mørkve et al, 1992). In comparison, in one immunohistochemical study of pancreatic carcinoma, the proliferating index (percentage of cells > $G_1$ $G_0$) was not associated with p53 immunoreactivity (DiGuiseppe et al, 1994). It has been suggested that Ki-67 is not involved in the same steps of cell proliferation as p53, and therefore a similar staining pattern could not be expected (Thompson et al, 1992).

In conclusion, immunohistochemical staining by the MIB-1 antibody is feasible in paraffin sections of pancreatic carcinoma. The pattern of expression is clear and is possible to evaluate by conventional scoring. Alternatively, it is possible to calculate the PCI and PCA using an interactive image analysis system. In the majority of tumours, expression of p53 protein can be evaluated using the immunohistochemical technique. Although the number of patients in the present series is limited, highly proliferative pancreatic tumours or those expressing the p53 protein were associated with shorter survival time.

### ACKNOWLEDGEMENTS

This work was supported by grants from Swedish Medical Research Council (project no. 102), the Swedish Cancer Society (project no. 2841), the Cancer Society of Stockholm, the King Gustav V Jubilee Fund, Stockholm, the Research Funds of the Faculty of Medicine at the Karolinska Institute, Stockholm, and the Swedish Society of Medicine (the Bengt Ihre Fund). A visiting scientist research fellowship was received by CP both from the Swedish Institute in Stockholm and from the Ministry of Education, Spain. We would also like to thank Mr Bo Nilsson, Department of Cancer Epidemiology and Biostatistics, Karolinska Hospital, Stockholm, for performing the statistical calculations.

### REFERENCES

Barton CM, Staddon SL, Hughes CM, Hall PA, O'Sullivan C, Klöppel G, Theis B, Russel RCG, Neoptolemos J, Williamson RCN, Lane DP and Lemoine NR (1991) Abnormalities of the p53 tumour suppressor gene in human pancreatic cancer. *Br J Cancer* **64**: 1076–1082

Baas IO, Van Den Berg FM, Mulder J-WR, Clement MJ, Slebos RJC, Hamilton SR and Offerhaus GJA (1996) Potential false-positive results with antigen enhancement for immunohistochemistry of the p53 gene product in colorectal neoplasms. *J Pathol* **178**: 264–267

Van Den Berg FM, Baas IO, Polak MM and Offerhaus GJA (1993) Detection of p53 overexpression in routinely paraffin-embedded tissue of human carcinomas using a novel target unmasking fluid. *Am J Pathol* **142**: 381–385

Berrozpe G, Schaeffer J, Peinado MA, Real FX and Perucho M (1994) Comparative analysis of mutations in the p53 and K-ras genes in pancreatic cancer. *Int J Cancer* **58**: 185–191

Bodner SM, Minna JD, Jensen SM, D'Amico D, Carbone D, Mitsudomi T, Fedorko J, Buchhagen DL, Nau MM, Gazdar AF and Linnoila RI (1992) Expression of mutant P53 proteins in lung cancer correlates with the class of p53 gene mutation. *Oncogene* **7**: 743–749

Digiuseppe JA, Hruban RH, Goodman SN, Polak M, Van Den Berg FM, Allison DC, Cameron JL and Offerahaus JA (1994) Overexpression of p53 protein in adenocarcinoma of the pancreas. *Am J Clin Pathol* **101**: 684–688

Hamelin R, Laurent-Puig P, Olschwang S, Jege N, Asselain B, Remvikos Y, Girodet J, Salomon RJ and Thomas G (1994) Association of p53 mutations with short survival in colorectal cancer. *Gastroenterology* **106**: 42–48

Hermanek P and Sobin LH (1987) *UICC International Union Against Cancer. TNM Classification of Malignant Tumors*, 4th fully revised edn. Springer-Verlag: Berlin

Isola J, Visakorpi T, Holli K and Kallioniemi OP (1992) Association of overexpression of tumor suppressor protein p53 with rapid cell proliferation and poor prognosis in node-negative breast cancer patients. *J Natl Cancer Inst* **84**: 1109–1114

Kalthoff H, Schmiegel W, Roeder C, Kasche D, Schmidt A, Lauer G, Thiele H-G, Honold G, Pantel K, Riethmüller G, Scherer E, Maurer J, Maacke H and Deppert W (1993) p53 and K-RAS alterations in pancreatic epithelial cell lesions. *Oncogene* **8**: 289–298

Kelleher L, Magee HM and Dervan PA (1994) Evaluation of cell-proliferation antibodies reactive in paraffin sections. *Appl Immunohistochem* **2**: 164–170

Lam K-Y, Law S Y-K, So M K-P, Fok M, Ma LT and Wong J (1996) Prognostic implication of proliferative markers MIB-1 and PC10 in esophageal squamous cell carcinoma. *Cancer* **77**: 7–12

 © Cancer Research Campaign 1997

Linder S, Lindholm J, Falkmer U, Blåsjö M, Sundelin P and Von Rosen A (1995) Combined use of nuclear morphometry and DNA ploidy as prognostic indicators in nonresectable adenocarcinoma of the pancreas. *Int J Pancreatol* **18**: 241–248

Lundin J, Nordling S, Von Boguslawsky K, Roberts PJ and Haglund C (1995) Prognostic value of Ki-67 expression, ploidy and S-phase fraction in patients with pancreatic cancer. *Anticancer Res* **15**: 2659–2668

Lundin J, Nordling S, Von Boguslawski K, Roberts PJ and Haglund C (1996) Prognostic value of immunohistochemical expression of p53 in patients with pancreatic cancer. *Oncology* **53**: 104–111

Martin HM, Filipe MI, Morris RW, Lane DP and Silvestre F (1992) p53 expression and prognosis in gastric carcinoma. *Int J Cancer* **50**: 859–862

McCormick D, Chong H, Hobbs C, Datta C and Hall PA (1993) Detection of the Ki-67 antigen in fixed and wax-embedded sections with the monoclonal antibody MIB1. *Histopathology* **22**: 355–360

Mørkve O, Halvorsen OJ, Stagenland L, Gulsvik A and Laerum OD (1992) Quantitation of biological tumor markers (p53, c-myc, Ki-67 and DNA-ploidy) by multiparameter flow cytometry in non-small-cell lung cancer. *Int J Cancer* **52**: 851–855

Parrado C, Falkmer UG, Höög A, Falkmer S, Ahrens O, Rius F and Grimelius L (1996) A technique for automatic/interactive assessment of the proliferating fraction of neoplastic cells in solid tumours. A methodological study on the zKi-67 immunoreactive cells in human mammary carcinomas, including a comparison with the results of conventional S-phase fraction assessments by means of DNA cytometry. *Gen Diagn Pathol* **141**: 215–227

Pellegata NS, Sessa F, Renault B, Bonato M, Leone BE, Solcia E and Ranzani GN (1994) K-ras and p53 gene mutations in pancreatic cancer: ductal and nonductal tumors progress through different genetic lesions. *Cancer Res* **54**: 1556–1560

Pinder SE, Wencyk P, Sibbering DM, Bell JA, Elston CW, Nicholson R, Robertson JFR, Blamey RW and Ellis IO (1995) Assessment of the new proliferation marker MIB1 in breast carcinoma using image analysis: associations with other prognostic factors and survival. *Br J Cancer* **71**: 146–149

Railo M, Nordling S, Von Boguslawsky K, Leivonen M, Kyllönen L and Von Smitten K (1993) Prognostic value of Ki-67 immunolabelling in primary operable breast cancer. *Br J Cancer* **68**: 579–583

Ruggeri B, Zhang SY, Caamano J, Dirado M, Flynn SD and Klein-Szanto AJP (1992) Human pancreatic carcinomas and cell lines reveal frequent and multiple alterations in the p53 and Rb-1 tumor-suppressor genes. *Oncogene* **7**: 1503–1511

Scarpa A, Capelli P, Mukai K, Zamboni G, Oda T, Iacono C and Hirohashi S (1993) Pancreatic adenocarcinomas frequently show p53 gene mutations. *Am J Pathol* **142**: 1534–1543

Thompson SJ, Mellon K, Charlton RG, Marsh C, Robinson M and Neal DE (1992) P53 and Ki-67 immunoreactivity in human prostate cancer and benign hyperplasia. *Br J Urol* **69**: 609–613

Weidner N, Moore DH and Vartanian R (1994) Correlation of Ki-67 antigen expression with mitotic figure index and tumor grade in breast carcinomas using the novel 'paraffin'-reactive MIB1 antibody. *Hum Pathol* **25**: 337–342

© Cancer Research Campaign 1997